# Calcium Dependent CAMTA1 in Adult Stem Cell Commitment to a Myocardial Lineage

**DOI:** 10.1371/journal.pone.0038454

**Published:** 2012-06-08

**Authors:** Barbara Muller-Borer, Gwyn Esch, Rob Aldina, Woohyun Woon, Raymond Fox, Nenad Bursac, Sylvia Hiller, Nobuyuo Maeda, Neal Shepherd, Jian Ping Jin, Mary Hutson, Page Anderson, Margaret L. Kirby, Nadia N. Malouf

**Affiliations:** 1 Department of Pathology & Laboratory Medicine, University of North Carolina at Chapel Hill, Chapel Hill, North Carolina, United States of America; 2 Department of Pediatrics, Duke University, Durham, North Carolina, United States of America; 3 Department of Cell Biology, Duke University, Durham, North Carolina, United States of America; 4 Department of Biomedical Engineering, Duke University, Durham, North Carolina, United States of America; 5 Department of Cardiovascular Sciences, East Carolina University, Greenville, North Carolina, United States of America; 6 Center for Bioinformatics, University of North Carolina at Chapel Hill, Chapel Hill, North Carolina, United States of America; University of California, Merced, United States of America

## Abstract

The phenotype of somatic cells has recently been found to be reversible. Direct reprogramming of one cell type into another has been achieved with transduction and over expression of exogenous defined transcription factors emphasizing their role in specifying cell fate. To discover early and novel endogenous transcription factors that may have a role in adult-derived stem cell acquisition of a cardiomyocyte phenotype, mesenchymal stem cells from human and mouse bone marrow and rat liver were co-cultured with neonatal cardiomyocytes as an in vitro cardiogenic microenvironment. Cell-cell communications develop between the two cell types as early as 24 hrs in co-culture and are required for elaboration of a myocardial phenotype in the stem cells 8–16 days later. These intercellular communications are associated with novel Ca^2+^ oscillations in the stem cells that are synchronous with the Ca^2+^ transients in adjacent cardiomyocytes and are detected in the stem cells as early as 24–48 hrs in co-culture. Early and significant up-regulation of Ca^2+^-dependent effectors, CAMTA1 and RCAN1 ensues before a myocardial program is activated. CAMTA1 loss-of-function minimizes the activation of the cardiac gene program in the stem cells. While the expression of RCAN1 suggests involvement of the well-characterized calcineurin-NFAT pathway as a response to a Ca^2+^ signal, the CAMTA1 up-regulated expression as a response to such a signal in the stem cells was unknown. Cell-cell communications between the stem cells and adjacent cardiomyocytes induce Ca^2+^ signals that activate a myocardial gene program in the stem cells via a novel and early Ca^2+^-dependent intermediate, up-regulation of CAMTA1.

## Introduction

It has become well recognized that transcription factors have a crucial role in reprogramming gene expression in mammalian cells and that the process of cell differentiation can be reversed [1], [2], [3], [4], [5], [6], [7], [8], [9], [10], [11], [12], [13], [14], [15], [16], [17]. Differentiated somatic cells from various tissues and species including humans have been reprogrammed into pluripotency by transduction and over expression of defined transcription factors [2], [4], [5], [7], [10]. More recently direct reprogramming of one cell type into another, without resorting to an intermediate pluripotent stage has been achieved with over-expression of tissue specific transcription factors [13], [14], [15], [16], [17]. These findings raise the possibility that targeted manipulation of a less stringent epigenetic restrictive state in multipotent adult-derived stem cells may be achieved, so as to induce the endogenous expression of a transcriptional program that characterizes a specific cell fate.

As the molecular basis underlying adult-derived stem cell commitment to a myocardial lineage is poorly understood [18], [19], [20], [21], [22] we attempted in the present study to identify novel and early transcription factors that activate the expression of a myocardial transcriptional program in the stem cells without the introduction of exogenous genetic material [13], [14], [15], [16], [17]. We have previously shown that cells from a cloned rat liver stem cell line (WB F344) acquired a cardiac phenotype in vivo and when co-cultured with rat neonatal cardiomyocytes as an in vitro cardiogenic microenvironment [23], [24], [25]. Using fluorescence recovery after photobleaching (FRAP) we found that the stem cell-derived nascent cardiomyocytes were functionally coupled with adjacent cardiomyocytes through gap junctions. This is associated with novel Ca^2+^ oscillations that are synchronous with Ca^2+^ transients in adjacent cardiomyocytes and detected in the stem cells as early as 24–48 hrs in co-culture with the cardiomyocytes.

Since evidence suggests that intracellular Ca^2+^ signals trigger transcriptional responses and that the diversity of responses in different cell types results from the variability in the frequency and duration of the Ca^2+^ signals [26], [27], [28], [29], [30], [31], [32], [33], [34], [35], [36], [37], we explored the possibility that these novel Ca^2+^ signals may be decoded in bone marrow mesenchymal stem cells from human (hMSCs) and mouse (mMSCs) by activating a cardiac gene program.

We find that the expression of the transcription factor CAMTA1, a member of a recently recognized family of Ca^2+^-dependent calmodulin binding transcription activators conserved in eukaryotes [38], [39], [40], [41], [42] and RCAN1, a known regulator of calcineurin [36], [43], [44], [45], to be significantly up-regulated in the stem cells as early as 24 hrs in co-culture with rat neonatal cardiomyocytes. This process preceded stem cell acquisition of other cardiac properties. Cardiac specific transcription factors appear after 2–4 days in co-culture and myocardial contractile proteins 8–16 days later. Furthermore, the expression of CAMTA1 was up-regulated in the stem cells cultured alone when exposed to ionomycin, which produces a tonic increase in intracellular Ca^2+^ ([Ca^2+^]_i_). This was associated with novel expression of cardiac transcription factors in the stem cells. When CAMTA1 expression is suppressed with CAMTA1 siRNA or shRNA before the stem cells are exposed to an increase in [Ca^2+^]_i_ the expression of the corresponding cardiac transcription factors is significantly decreased.

Collectively, our results support the hypothesis that [Ca^2+^]_i_ signals in adult-derived stem cells relay messages that activate downstream pathways where up-regulation of CAMTA1 expression, is an early event in the stem cell commitment to a myocardial lineage.

## Results

### Three Different Types of Adult-Derived Stem Cells Acquire Cardiomyocyte Properties in Co-Culture with Cardiomyocytes

**Figure 1:**
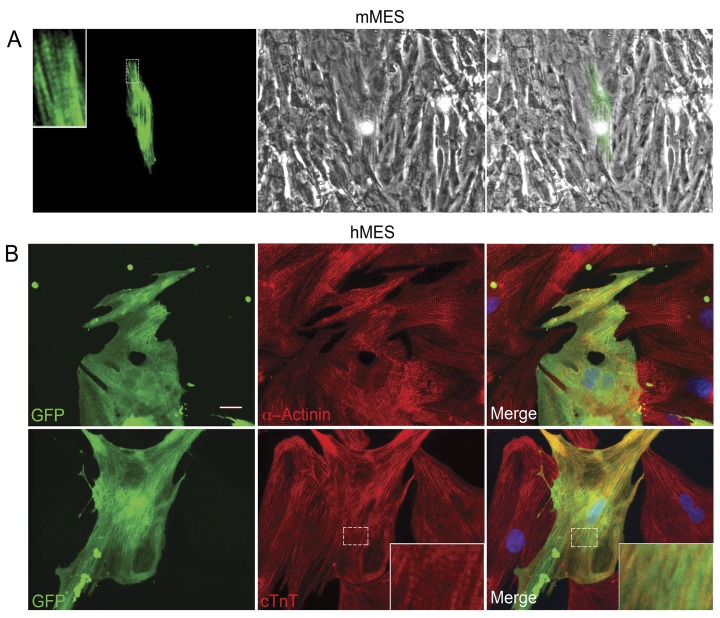
Bone marrow-derived MSCs in co-culture with cardiomyocytes. (A) A nascent cardiomyocyte derived from mMSCs of a βMHC-YFP genotype mouse at 8 days in co-culture with cardiomyocytes. The βMHC-YFP cell is pseudo colored green for visualization of the striations. Left panel shows novel endogenous expression of βMHC-YFP fluorescence along stress fibers and striations (magnified in insert). Middle panel shows surrounding rat cardiomyocyte. Merged images in right panel. (B) GFP-hMSCs co-cultured with cardiomyocytes for 16 days and immunostained for α-actinin or troponin T ([23] and Supporting Information S1). Acquisition of a differentiated cardiac phenotype is demonstrated in the inserts where cardiomyocyte striations are visible. Nuclear DAPI in blue. Scale bar = 20 µm.

In spite of the focus of this study on the earliest transcriptional events, we nevertheless show that bone marrow mesenchymal stem cells from hMSCs and mMSCs acquire a cardiac phenotype in co-culture with cardiomyocytes similar to our previous report on rat liver stem cells (WB F344) [23], [24], [25]. Expression of a differentiated cardiac phenotype where contractile proteins are localized in a striated pattern is demonstrated in the stem cells after 8–16 days in co-culture with rat neonatal cardiomyocytes (Figure 1). Human MSCs constitutively labeled with GFP in the cytoplasm or dsRed targeted to the mitochondria and mMSCs from C57BL/6 mice with the following genotypes: a beta Myosin Heavy Chain (βMHC)-YFP fusion transgenic mouse and αMHC-CFP/βMHC-GFP transgenic mouse [46], [47] were prepared. Mouse MSCs were isolated from the bone marrow of the mice as previously reported [48], [49]. The mMSCs demonstrated no fluorescence until after 8 days in co-culture, at which time few cells exhibited spontaneous faint fluorescence indicating the onset of expression of the MHC gene in these cells. The intensity of the fluorescence and the number of fluorescent cells increased over the next 7 days. By two weeks, elongated fluorescent cells were seen adjacent to beating rat cardiomyocytes (Figure 1). In the case of the mMSCs derived from the βMHC-YFP mouse the corresponding nascent cardiomyocytes expressed novel yellow fluorescence along striations and stress fibers (Figure 1A). In contrast, the alpha MHC-CFP fluorescence from the corresponding mouse was cytoplasmic as the MHC-CFP gene was engineered as a single copy in the HPRT locus (data not shown). Once dissociated and harvested using FACS, the mMSCs population of cells that expressed YFP-βMHC represented a relatively pure population of mMSCs that had undergone “differentiation”. These cells were used to monitor the efficiency of the differentiation process (10–15%) calculated as a fraction of the number of non-fluorescent native mMSCs originally seeded on the neonatal rat cardiomyocytes.

The hMSCs pre-labeled with GFP or dsRed started expressing cardiac specific troponin T (cTnT) and alpha-actinin after 8–16 days in co-culture. These proteins could be seen by immunocytochemistry along striations and stress fibers of differentiating GFP-labeled hMSCs (Figure 1B). The number of GFP-labeled hMSCs in co-cultures that demonstrated expression of cTnT or alpha-actinin proteins by immunocytochemistry was calculated to be 21±0.2% (n = 3).

To confirm previous reports by us and others [23], [50], [51], [52], [53] that cell-cell contact with cardiomyocytes is the most likely initiator of differentiation of stem cells into a cardiac lineage we ruled out a role for paracrine or juxtacrine factors from the neonatal rat cardiomyocytes. Native hMSCs in monocultures which were exposed to a neonatal rat cardiomyocyte lysate showed no up-regulation in the expression of CAMTA1 or cardiac transcription factors (data not shown). Furthermore, the combination of growth factors that has been shown to enhance differentiation of embryonic stem cells into cardiomyocytes [54], [55], [56] did not induce any of the adult-derived stem cell lines to differentiate into cardiomyocytes (data not shown). Fusion with cardiomyocytes as the only mechanism for the stem cells acquisition of a cardiomyocyte phenotype was ruled out in stem cell monoculture studies (see below).

### Ca^2+^ Oscillations in hMSCs Co-Cultured with Cardiomyocytes

**Figure 2:**
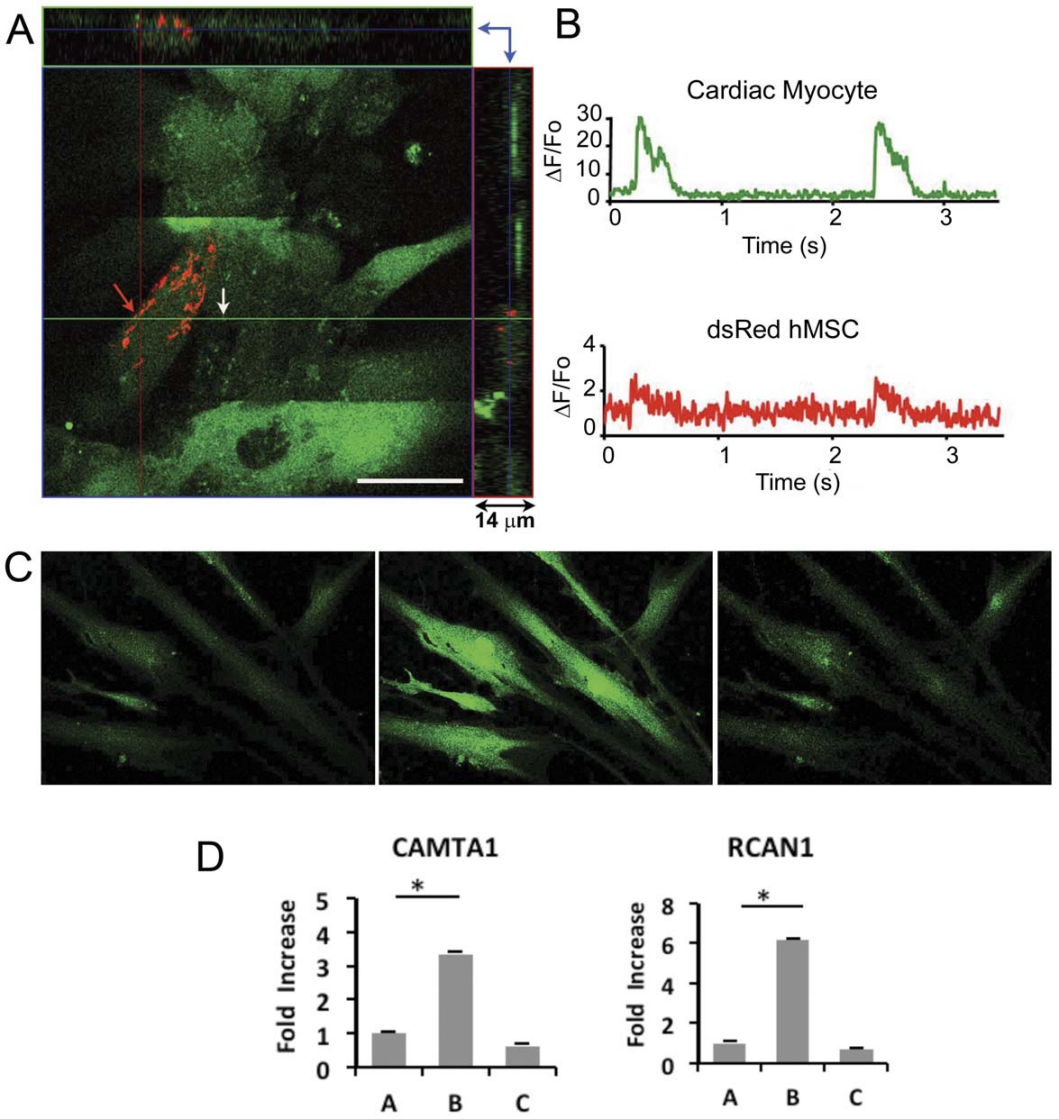
Intracellular Ca^2+^ signals acquired from a dsRed hMSC and neonatal cardiac myocyte, after 48 hrs in co-culture. (A) Illustrates the three dimensional distribution of hMSCs and cardiac myocytes in co-culture. The image shows a 1.0 µm confocal optical section from a 14 µm thick co-culture. The section shown corresponds to the regions indicated by the blue arrows, confirming that the cells were not overlapping (right upper quadrant). A line scan was acquired along the green horizontal line. Scale bar = 20 µm. (B) Using confocal line scan microscopy Ca^2+^ oscillations corresponding to the hMSCs (red arrow in A) and a cardiac myocyte (white arrow in A) were recorded. Top panel shows Ca^2+^ oscillations recorded in the cytosol of a cardiac myocyte, noted by the white arrow in A. Bottom panel shows perinuclear Ca^2+^ oscillation recorded from a young dsRed hMSC adjacent to a cardiac myocyte (red arrow in A). Note the difference in Ca^2+^ signal amplitude at this early time point. Ca^2+^ signals were recorded on a Zeiss laser scanning confocal microscope at room temperature. All cells were labeled with Fluo-4 AM. Cardiac myocytes were beating spontaneously. (C) Human MSCs response to ionomycin. Fluo-4 fluorescence measured in naive hMSCs in monoculture (left panel), 10 sec after the application of 1 µMol/L ionomycin (middle panel), and 60 sec after the application of 2 mMol/L EGTA (right panel). (D) Transcriptional response to ionomycin-induced intracellular calcium in hMSCs in monocultures. Human MSCs in monocultures were stimulated for 6 hrs with 0.6 µMol/L ionomycin. RT-qPCR from RNA harvested from A: Untreated, control hMSCs. B: hMSCs after 6 hr ionomycin stimulation, C: hMSCs stimulated for 6 hrs with ionomycin, washed, cultured in fresh medium and harvested after 24 hrs of ‘recovery’. Expression of CAMTA1 (left panel); RCAN (right panel). The bars show mean ± SEM, *p<0.05.

Line scanning confocal fluorescence microscopy was performed to record intracellular Ca^2+^ signals in hMSCs adjacent to cardiomyocytes in co-culture. Synchronous Ca^2+^ oscillations were detected in the stem cells that were adjacent to a neonatal cardiomyocyte as early as 24–48 hrs in the cytoplasm of approximately 1 in 10–20 hMSCs cardiomyocyte (Figure 2). The location and origin of each Ca^2+^ recording was verified by acquiring a 3D Z stack image through the cell’s depth. Ca^2+^ recordings were acquired from hMSCs and cardiomyocytes that were not overlapping. The hMSC Ca^2+^ oscillations were of the same frequency but lower amplitude than the Ca^2+^ transients in adjacent spontaneously contracting or paced cardiomyocytes. The duration of the novel Ca^2+^ oscillations in the hMSCs was 375– 400 ms, significantly shorter than the slow, endogenous Ca^2+^ transients in MSCs cultured alone [57]. Ca^2+^ oscillations were not detected in hMSCs seeded away from cardiomyocytes in the co-culture. Consistent with our findings in WB F344 cells co-cultured with cardiomyocytes [23], [25], these results suggest that cell-cell communication between hMSCs and cardiomyocytes might trigger the acquisition of oscillating Ca^2+^ signals in the stem cells.

### Transcriptional Profiling of Co-Cultured Stem Cells

**Figure 3:**
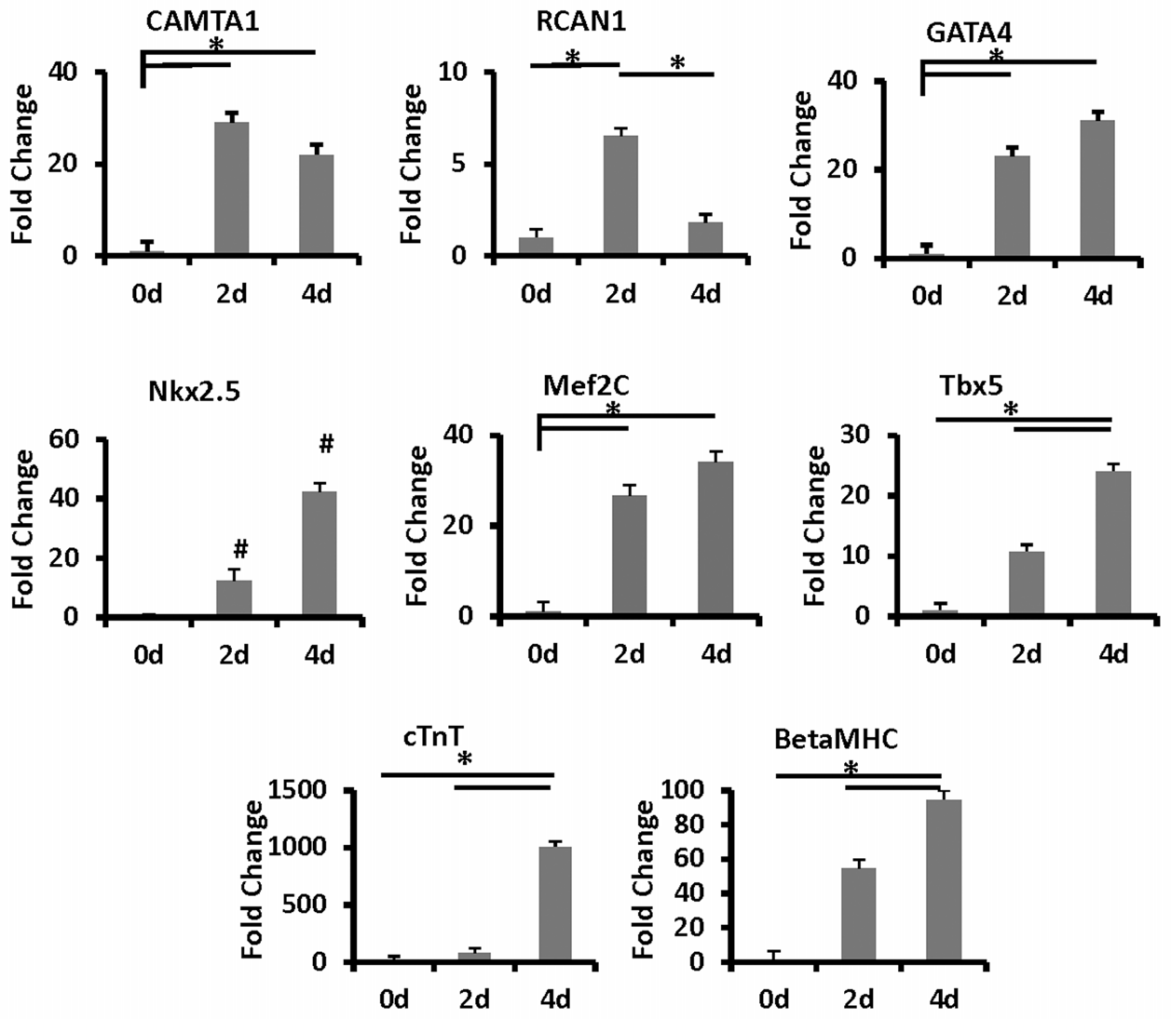
Time course of RNA expression levels of cardiac transcription factors and contractile protein genes in hMSCs co-cultured with rat neonatal cardiomyocytes. Control levels of CAMTA1, RCAN1, GATA4, Nk×2.5, Mef2c, Tb×5, cTnT, and BetaMHC in the stem cells in monoculture at day 0 (0d), or co-cultured with cardiomyocytes for 2 days(2d) and 4 days (4d). The bars show mean ± SEM, *p<0.05. # denotes novel expression in Nkx2.5.

To uncover early transcription factors that have a role in stem cell commitment to a cardiomyocyte lineage, we performed a screening transcriptional expression microarray on hMSCs co-cultured for 4 days with cardiomyocytes, well before they acquired recognizable cardiac properties 8–16 days later. DsRed hMSCs were separated from the non-labeled cardiomyocytes using trypsinization buffers, and harvested with fluorescence activated cell sorting (FACS, see Methods). The RNA for the microarray analysis was prepared from three biologically separate samples of hMSCs co-cultured with cardiomyocytes for 4 days. The results from the microarray (NCBI-GEO data base accession number GSE32171) demonstrated significant up-regulation in the expression of two Ca^2+^-dependent factors, CAMTA1 and RCAN1. The expression of CAMTA1 was negligible in naive hMSCs but showed a significant 5.5 fold up-regulation (p≤0.0321) after they were co-cultured with cardiomyocytes. In contrast, CAMTA2, expressed a priori in the hMSCs, did not significantly change when the stem cells were co-cultured with cardiomyocytes. The microarray also showed that the expression of RCAN1 was significantly up-regulated by 4 fold (p≤0.008) in the hMSCs 4 days after they were co-cultured with neonatal cardiomyocytes (Figure 2D). As the focus of this study was to investigate the role of CAMTA1 in the early induction of cardiac transcription factors expression in adult-derived stem cells, we did not examine at this stage, the hierarchal relationship between CAMTA1 and RCAN1 in this developmental process. Rather, we used and monitored the expression of RCAN1 along with that of CAMTA1 as a reference for a Ca^2+^ signal response in the stem cells. RCAN1 is a direct target of the Ca^2+^/calcineurin-NFAT signaling pathway [43] and its up-regulation suggests involvement of this signaling pathway in the hMSCs co-cultured with cardiomyocytes. The results of the microarray were confirmed by RT-qPCR using RNA from dsRed or GFP-labeled hMSCs and from dsRed-labeled liver stem cells. As both cell types expressed fluorescent proteins, they were FACS harvested after 2 and 4 days in co-culture with neonatal rat cardiomyocytes (Figure 3). Using species specific primers (Table S2) the reactions were carried out in triplicate on RNA isolated from sorted fluorescent stem cells in co-cultures.

**Figure 4:**
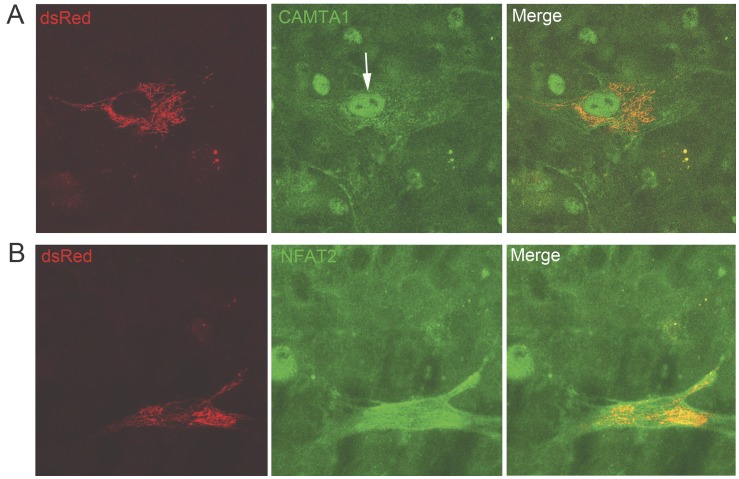
Protein localization by immunocytochemistry in hMSCs co-cultured with cardiomyocytes for 48 hours. Left panels show dsRed-hMSCs, middle panels show CAMTA1 or NFAT protein localization. Right panels show merged images. (A) Novel expression of CAMTA1 protein is demonstrated in the nucleus of a dsRed hMSCs co-cultured with rat cardiomyocytes (arrow in middle panel). Rat cardiomyocytes (small nuclei in the background) a priori express CAMTA1. Note the larger human nucleus of the hMSCs compared to the size of the rat nuclei in the background. (B) Novel NFAT2c protein expression in an hMSC co-cultured with cardiomyocytes. NFAT2c is localized throughout the hMSC including the nucleus of the hMSC. NFAT2c was not detected in the nucleus in naïve hMSCs.

RT-qPCR was also used to monitor the expression of cardiac specific transcription factors and contractile protein genes and the early progression of the stem cell commitment and their differentiation to a myocardial lineage (Figure 3). Whereas the expression of CAMTA1 and RCAN1 reached their peak expression at about 24 hr in co-culture, the expression of other cardiac specific transcription factors, Nk×2.5, Tb×5, Mef2C and GATA4 and the contractile protein genes, βMHC and cTnT began to rise soon after and continued to increase 2 days later (Figure 3). These results suggest that signaling through CAMTA1 and indirectly Ca^2+^/calcineurin/NFAT may be early events in the initiation of stem cell commitment into a cardiomyocyte phenotype. Up-regulation in the expression of CAMTA1 and RCAN1 was associated with nuclear localization, demonstrated by immunocytochemistry of CAMTA1 and NFAT2C proteins in the co-cultured hMSCs (Figure 4). NFAT in combination with other transcription factors is believed to orchestrate expression of cardiogenic and other developmental genes [36], [43], [44], [45], [58], [59], [60], [61], [62] and our data indicates that CAMTA1, in adult-derived stem cells, contributes to this process.

We queried the microarray as to the status of the “pluripotent” factors [4], [5], [10]: Oct4, Sox2, c-Myc, and KLF4, LIN28 and NANOG to determine whether the stem cells underwent reprogramming into a pluripotent state before they acquired a myocardial fate. With the exception of c-Myc, none of these factors were significantly changed in the hMSCs co-cultured with cardiomyocytes. On the other hand, as reprogramming of stem cells is reported to be associated with chromatin remodeling [14], [63], [64], [65], [66], [67], [68] the expression of BRG1, BRM and BAF 57, members of the SWI/SNF chromatin remodeling complex, was significantly downregulated in hMSCs co-cultured for 4 days with cardiomyocytes suggesting that these adult-derived stem cells underwent a relative withdrawal from an unstable “stemness” state (Table S1).

### Increased Intracellular Ca^2+^ in Stem Cells Cultured Alone Leads to Up-regulated Expression of Transcription Factors

The role of increased [Ca^2+^]_i_ on the expression of transcription factors was examined in all three types of stem cells in monocultures, without the benefit associated with co-cultured cardiomyocytes. The [Ca^2+^]_i_ increase was induced with 0.6 µMol/L of the Ca^2+^ ionophore, ionomycin added to the culture medium. The increase in global Ca^2+^ concentration was monitored with the fluorescent Ca^2+^- sensitive fluoroprobe, Fluo-4 (Figure 2C). Under this condition, the expression of CAMTA1 and RCAN1 was significantly up-regulated within 6 hrs after introduction of the ionophore in all three types of stem cells (Figure 2D). Interestingly, the transcriptional level of other family members namely, CAMTA2 and RCAN3 was unchanged under these conditions, suggesting their independence from Ca^2+^ signaling in the stem cells. Twenty-four hours after termination of the Ca^2+^ signaling the expression of these transcription factors returned to baseline levels (Figure 2D). We attribute the sustained expression of these factors in the stem cells co-cultured with cardiomyocytes to the continuous Ca^2+^ oscillations in the stem cells while in physical contact with cardiomyocytes.

**Figure 5:**
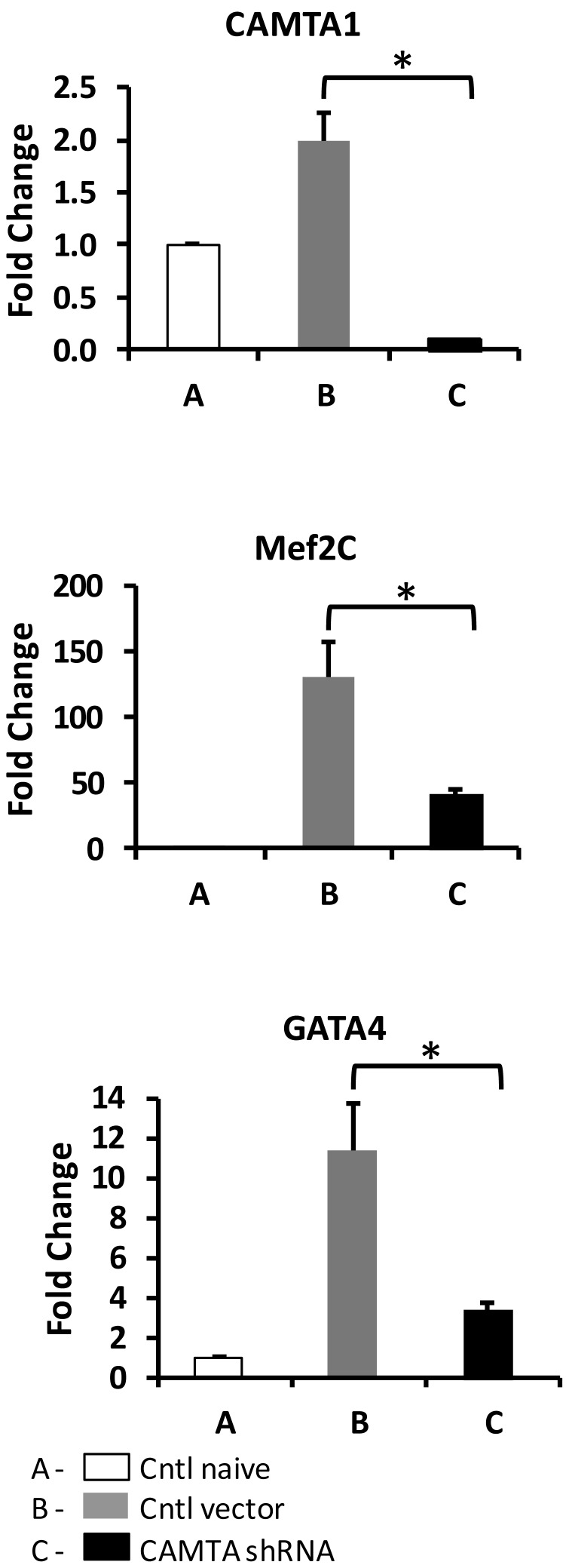
Regulation of expression of cardiac transcription factors following the minimization of CAMTA1 expression in hMSCs monocultures. Human MSCs were pre-transduced with specific lentiviral vectors 24 hrs before the up-regulation of CAMTA1 expression was induced with ionomycin. A: control, naive hMSCs grown in monoculture, B: hMSCs pre-transduced with a GFP-lentiviral vector, then stimulated with ionomycin. C: hMSCs pre-transduced with CAMTA1 shRNA lentiviral vector, then stimulated with ionomycin. The graphs illustrate expression of the transcription factors CAMTA1, Mef2C, and Gata4. Note condition B shows increased expression of Mef2C and Gata 4 with increased CAMTA1 expression. With minimization of CAMTA1 expression (condition C) expression of Mef2C and Gata4 was significantly decreased (see Figure 2D for CAMTA1 control). The bars show mean ± SEM, *p<0.05.

The expression of the transcription factors Mef2C and GATA4, was also up-regulated with increase in [Ca^2+^]_i_ in the MSCs in monocultures exposed to ionomycin (Figure 5). The latter finding of Ca^2+^-dependent up-regulation in the expression of the cardiac transcription factors Mef2C and GATA4 in the hMSC in monocultures argues against fusion of the stem cells with surrounding cardiomyocytes as the only mechanism for adult-derived stem cell commitment to myocardial lineage.

### A Role for CAMTA1 in Stem Cell Differentiation

That CAMTA1 has a role in stem cell commitment to a cardiac lineage was investigated in loss-of function studies. Human CAMTA1 shRNA lentiviral particles were pre-transduced in the hMSCs to minimize the ionomycin/Ca^2+^-induced up-regulation of CAMTA1 expression. These studies were first established in hMSC monocultures where CAMTA1 expression could be acutely induced with ionomycin (Figure 2D and Figure 5, CAMTA 1, condition B). Two days after the stem cells in monocultures were pre-transduced with CAMTA1 shRNA they were exposed to 0.6 µMol/L ionomycin for 6 hrs. RNA was isolated from all samples for RT-qPCR. HMSCs pretreated with CAMTA1 shRNA showed attenuated expression of Mef2C and GATA4 as well as that of CAMTA1 after ionomycin treatment (Figure 5).

**Figure 6:**
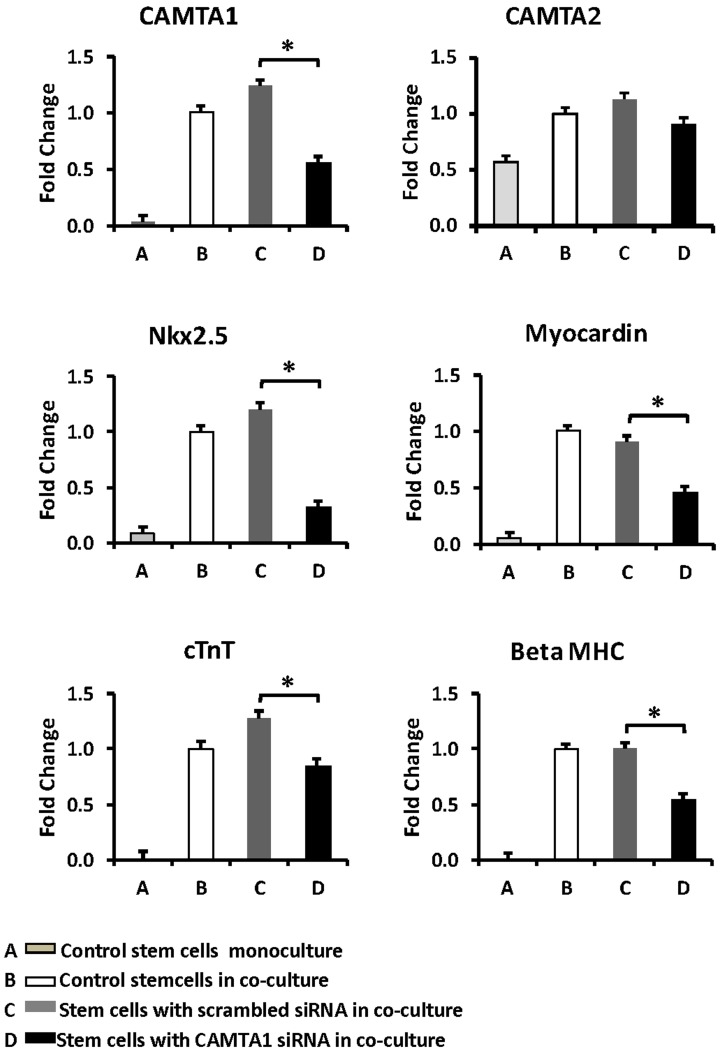
Regulation of cardiac transcription factors in liver stem cells co-cultured with cardiomyocytes after minimizing CAMTA1 expression. Except for the control conditions (A and B) the stem cells were pre-transfected with specific siRNA 24 hrs before they were added to the co-culture with cardiomyocytes. A: control, naive stem cells in monoculture for 72 hrs. B: naive stem cells cultured for 24 hrs, then co-cultured with cardiomyocytes for 48 hrs. C: Stem cells pre-transfected with a scrambled siRNA 24 hrs before they were co-cultured for 48 hrs with cardiomyocytes. D: Stem cells were pre-transfected with a human pool of CAMTA1 siRNAs for 24 hrs before they were co-cultured for 48 hrs with cardiomyocytes. The graphs illustrate expression of the transcription factors CAMTA1, CAMTA2, Nk×2.5, Myocardin, cTnT and BetaMHC. A significant decrease in the expression of transcription factors Nkx2.5, Myocardin, cTnT and BetaMHC were measured when CAMTA1 expression was minimized. Note that no significant change in CAMTA2 expression was observed. The bars show mean ± SEM, *p<0.05.

As the hMSCs did not survive long enough in coculture to allow activation of a cardiac gene program (i.e. 4 days) after they were transduced with the transfecting reagents, we used the liver stem cells (WB F344) to examine the effects of minimizing CAMTA1 expression on activation of a cardiac program in co-cultured stem cells. This cloned rat liver stem cell line is more resilient to transfection and the activation of a cardiac program is earlier (2 days) than that in the hMSCs (greater than 4 days) in co-culture. DsRed WB F344 stem cells were pre-transfected with a pool of human CAMTA1 siRNAs before they were added to the cardiomyocytes. Only one of the human CAMTA1 siRNA sequences in the siRNA pool was identical to that of the rat. A generic GFP labeled fluorescent siRNA was used to calculate the transfection efficiency to be 80%. After two days in co-culture with cardiomyocytes, the pre-transfected stem cells were harvested for RNA extraction and RT-qPCR. CAMTA1 siRNA pre-transfected for 48 hrs in the dsRed stem cells caused significant decrease in CAMTA1 expression after they were co-cultured for 2 days with cardiomyocytes compared with stem cells that were co-cultured with cardiomyocytes but not pre-transfected with CAMTA1 siRNA (Figure 6). This was associated with down-regulation in expression of cardiac specific transcription factors, myocardin, and Nkx2.5 but not that of CAMTA2. The expression of the cardiac contractile genes, cTnT and βMHC was also decreased in the pre-transfected stem cells in co-culture (Figure 6). Together these results suggest an important role for CAMTA1 in the activation of a cardiac gene program in the stem cells.

## Discussion

Our study was designed to uncover new and early endogenous transcription factors that may have a role in directing adult-derived stem cell to activate a cardiac gene program. Our results suggest that a Ca^2+^- dependent signaling pathway that includes significant up-regulation in expression of CAMTA1 and RCAN1 is an early event in the induction of a myocardial gene program in the stem cells. We focused exclusively on CAMTA1 regulation by intracellular calcium concentration because of the novelty of our results in mammalian cells and similar findings in plants where the CAMTA1 gene product is believed to be an integrator between stress-induced intracellular Ca2+ increase and expression of genes that allow the plant to adapt to its environment [38], [39], [40], [41], [42]. We used the expression of RCAN1, also up-regulated in our system, to monitor a parallel response to a Ca2+ signal. While the role of the Ca^2+^/calcineurin/NFAT/RCAN1 pathway has been extensively studied in the adult heart and in development [30], [35], [36], [43], [44], [45], [58], [59], [60], [61], [69] the role for CAMTA1 proposed in embryonic cardiac development was never tested [42]. The CAMTA gene family is a family of activator/repressor transcription factors with a broad range of functions conserved in multicellular organisms [38], [39], [40], [41], [42]. As a response to a Ca^2+^ signal, the functional activity of CAMTA depends on its binding to the ubiquitous calmodulin in the cell, targeting downstream effectors, while its transcriptional role is determined through its nuclear interaction with other transcription factors [39] and a conserved CG-1 DNA binding domain [40], [41]. Two homologous CAMTA genes have been reported in mammalian heart [42]. CAMTA1, a putative tumor suppressor candidate [70], is suspected to have a role in embryonic cardiac development mediating the functions of transcription factors in the developing heart [42]. CAMTA2 shows expression after birth, is a potent co-activator of the cardiac specific transcription factor Nk×2.5, and is involved in cardiac hypertrophy [42]. The CAMTA1 allele in humans is located in the distal portion of the short arm of chromosome 1q3.6 [71], [72]. Spontaneous deletion of this subtelomeric chromosomal region in patients with the so-called “1p3.6 Deletion Syndrome” is associated with heart malformations and other cardiomyopathies in over 71% of cases [71], [72]. Coupled with our findings and those of Song et.al. [42] it is tempting to speculate that as a response to Ca^2+^ signaling, CAMTA1 may have a role in cardiogenesis in that deletion of the 1p36 chromosomal region may underlie the causation of cardiac abnormalities in some patients.

Interestingly, The CAMTA1 null mouse is embryonic lethal (Personal communication, Eric Olson) in contradistinction to that of RCAN1 (DSCR) where the null mouse exhibits no overt phenotype in the absence of stress [73]. The mechanisms underlying the lethality in the CAMTA1 null mouse are not known.

The authors queried the GEO Microarray Database attempting to elucidate a role for CAMTA1 in mammalian cells other than in the developing heart [42] and stem cell-derived cardiomyocytes as suggested by our findings. We found in the GEO Microarray Database (GDS814, GDS2387) that the expression of CAMTA1 is up-regulated and expressed as a sharp spike during the 1-cell stage embryo after fertilization of the oocyte. This coincides temporally with the well known increase in intracellular Ca^2+^ once the sperm fuses with the oocyte plasma membrane during fertilization [74]. Though a cause and effect are not linked in these reported studies, our present findings suggest that a single ionomycin-induced Ca^2+^ pulse in the stem cells cultured alone activates a transient expression of CAMTA1. Twenty four hours after termination of the Ca^2+^ signal the expression of CAMTA1 becomes down regulated to baseline levels (see Figure 2D). The sustained high level of CAMTA1 expression in the stem cells co-cultured over 4–16 days with cardiomyocytes is thought to be the result of continuous Ca^2+^ spikes oscillating in the stem cells while in physical contact with cardiomyocytes.

### Ca^2+^ Signal Response

Intracellular Ca^2+^ binds to calmodulin and activates among other pathways the Ca^2+^-dependent-calcineurin/NFAT signaling pathway [43], [69]. In a feedback loop NFAT activates the expression of RCAN1, an inhibitor of calcineurin, to protect the cells from unrestrained calcineurin activity [43], [44] where Ca^2+^ signals in the form of Ca^2+^ oscillations more efficiently elicit nuclear transcriptional responses [26], [27], [31], [37], [58]. Similarly our data suggest that as a response to Ca^2+^ signals in the stem cells, up-regulation of CAMTA1 expression contributed to the induction of the transcriptional program that directs a stem cell into a myocardial lineage [62], [75].

### Cell-Cell Communication

We and others have reported that cell-cell communication between the stem cells in co-culture with cardiomyocytes provides the necessary event/s including initiation of Ca^2+^ signaling that induce the stem cells to acquire a cardiac phenotype [23], [50], [51], [52], [53]. This is supported by our previously reported results showing that myocardial genes in the stem cells are not upregulated when the cardiomyocyte membranes are partially depolarized with the voltage-dependent Ca^2+^ channel antagonist, Nifedipine [23]. Nifedipine inhibits the Ca^2+^ transients in the cardiomyocytes, as well as the corresponding Ca^2+^ oscillations in the adjacent stem cells and is associated with a decrease in the expression of cardiac genes in the stem cells [23]. As reported by others [76] we examined the possibility that exocrine factors contributed to the differentiation of the mesenchymal stem cells into a cardiac lineage. We incubated the stem cells in a monoculture with a neonatal cardiomyocyte lysate. We found in this study as we reported previously [25] that naïve stem cells did not express CAMTA1 or cardiac transcription factors when exposed to a cardiac lysate or separated from the cardiomyocytes in culture with a semipermeable membrane. Yet, an intracellular Ca^2+^ signal induced with ionomycin in the hMSCs cultured alone resulted in the up-regulation of CAMTA1 and cardiac transcription factors (Figure 2D). Collectively, these findings support the hypothesis that novel Ca^2+^ oscillations in a population of stem cells co-cultured with cardiomyocytes are a result of physical contact between the membranes of the respective cell types and that these Ca^2+^ signals activate a downstream cardiogenic transcriptional response in the stem cells. To confirm that these findings can be independent of fusion between the stem cells and surrounding cardiomyocytes we ran parallel experiments (Figure 2D) where the hMSCs were cultured alone without the input of surrounding cardiomyocytes and exposed them to an ionomycin-induced/Ca^2+^ pulse. We found that hMSCs in monocultures respond to this Ca^2+^ signal by up-regulating the expression of CAMTA1 and cardiac transcription factors.

### Conclusion

Collectively, our results lead us to hypothesize that as a result of cell-cell membrane communication between the cardiomyocytes and the stem cells novel “cardiac-like” Ca^2+^ oscillations activate signaling pathways that result in a myocardial transcriptional program in the stem cells. CAMTA1 expression and activation of the calcineurin/NFAT/RCAN1 pathway are early and important events in the process. That the NFAT pathway alone was not sufficient to activate the cardiogenic transcriptional program in the stem cells is suggested in our silencing of CAMTA1 experiments which resulted in minimizing the expression of the cardiac specific transcription factors. Recently Li et al. [77] reported that the Ca^2+^-dependent calcineurin/NFAT signaling is critical for the early transition of mouse ES cells from self-renewal to lineage commitment. Though CAMTA1 expression was not investigated in that study, it is tempting to speculate that a response to Ca^2+^ signals may be a common process for lineage specification in stem cells.

## Methods

### Cardiac Microenvironment

The cardiac microenvironment was created as previously published [23]. Details are included in Supporting Information S1.

### Microarray

Microarrays were performed by the Functional Genomics Core Facility, Neuroscience Center at UNC, Chapel Hill, NC. Affymetrix Human U133 Plus 2.0 GeneChip array (Santa Clara, CA) was used. Seven mg of total RNA was used to synthesize cDNA. A custom cDNA kit from Life Technologies was used with a T7-(dT) 24 primer for this reaction. Biotinylated cRNA was then generated from the cDNA reaction using the BioArray High Yield RNA Transcript Kit. The cRNA was fragmented in fragmentation buffer (5X fragmentation buffer: 200 mMol/L Tris-acetate, pH 8.1, 500 mMol/L KOAc, 150 mMol/L MgOAc) at 94°C for 35 minutes before the chip hybridization. Fifteen mg of fragmented cRNA was then added to a hybridization cocktail (0.05 mg/ml fragmented cRNA, 50 pMol/L control oligonucleotide B2, BioB, BioC, BioD, and cre hybridization controls, 0.1 mg/ml herring sperm DNA, 0.5 mg/ml acetylated BSA, 100 mMol/L MES, 1 M [Na+], 20 mMol/L EDTA, 0.01% Tween 20). Ten mg of cRNA was used for hybridization. Arrays were hybridized for 16 hours at 45°C in the GeneChip Hybridization Oven 640. The arrays were washed and stained with R-phycoerythrin streptavidin in the GeneChip Fluidics Station 400. Arrays were scanned with the Hewlett Packard GeneArray Scanner. Affymetrix GeneChip Microarray Suite 5.0 software was used for scanning, and basic analysis. Sample quality was assessed by examination of 3′ to 5′ intensity ratios of certain genes.

### Microarray Data Analysis

The probe-level data from a set of Affymetrix CEL files were analyzed with GeneSpring GX 7.3.1 (Agilent Technologies, Englewood, CO). Only the perfect-match probe intensities were used for the robust multi-array analysis (RMA), which includes preprocesses of background correction, quantile normalization and probe set summarization. The background was estimated by using a kernel density estimation method. Quantile normalization of background corrected values across all arrays was performed. Finally median polishing, a robust model fitting technique, was applied for probe-set measurement summarization. For comparison of and viewing the data the preprocessed values were normalized to 50^th^ percentile for each array and then normalized to median values across all arrays for each gene. The principle component analysis and hierarchical clustering over test conditions and M-A plot were conducted for data quality estimation and control to ensure that no outlier array(s) was included in the analyzed data set. The probe sets with equal to or greater than 1.2 fold changes between the experimental conditions were kept for further analysis. Welch t-test was applied to the gene list. The differentially expressed gene list was created using Benjamini and Hochberg false discovery rate, with the p-value cut-off of 0.05 to reduce false positive discoveries.

### Sources of Adult-derived Stem Cells

The mouse bone marrow mesenchymal stem cells (mMSCs) were prepared from C57BL/6 mice with the following genotypes: a βMHC-YFP fusion mouse and αMHC-CFP/βMHC-GFP transgenic mouse (both provided by Dr. Kumar Pandya [46], [47]). MSCs were isolated as previously described [48], [49]. Briefly, 8–12 week old male mice were sacrificed by cervical dislocation, and the femurs and tibiae were removed. The tips of each bone was cut off, and the bone marrow was flushed out of the bone with Dulbecco’s Modified Eagle’s Medium (DMEM) containing 15% FBS. The isolated bone marrow was treated for 5 minutes at room temperature with ACK lysis buffer to remove red blood cells. The cells were filtered through a sterile 70 µm cell strainer. Cells were plated onto plastic 100 mm cell culture plates at a density of 10–20×10^6^ cells per plate. Plating medium consisted of DMEM, 15% FBS, 2 mm L-glutamine, β-mercaptoethanol, 100 u/ml penicillin, 100 u/ml streptomycin, and LIF. Cultures were maintained at 37°C in 95% air and 5% CO_2_. Passage 4–5 cells were used in the experiments. Approval of the procedures and methods to isolate mouse MSCs was obtained from the Institutional Animal Care and Use Committee of the University of North Carolina at Chapel Hill. Frozen vials of extensively characterized hMSCs from normal healthy donors were obtained from the Tulane Center for the Preparation and Distribution of Adult Stem Cells and Tulane Center of Gene Therapy under a Tulane University Institutional Review Board approved protocol through a grant from National Center for Research Resources of the US Department of Health and Human Services National Institutes of Health, Grant# p40RRO17447. The rat liver stem cells [23] were a gift from Dr. Joe Grisham at UNC at Chapel Hill. Approval of procedures and methods to isolate rat liver stem cells was obtained from the Institutional Animal Care and Use Committee of the University of North Carolina at Chapel Hill. All cells were prepared and co-cultured with rat neonatal cardiomyocytes as previously described [23].

### Immunocytochemistry

Immunocytochemistry was performed as previously reported [23] using the antibodies listed in Supporting Information S1.

### Real-time RT-PCR Amplification Method and Primer Design

Briefly, dsRed fluorescent hMSCs were separated from the non labeled cardiomyocytes using trypsinization buffers and sorted using a FACScan flow cytometer (Becton Dickinson, Franklin Lakes, NJ) equipped with a 70- or 100-µm nozzle, a 488-nm argon laser for excitation of the dsRed protein, and a 530±15-nm bandpass filter for monitoring fluorescent emission [23].

Total RNA was prepared from sorted dsRed hMSCs, dsRed WB F344 or YFP mMSCs using a Qiagen mRNA kit. For template preparation, 100–1000 ng of total RNA was transcribed to cDNA in the presence of 0.5 µg oligo dT, 0.25 mMol/L dNTPs, 1× First Strand Buffer and 200 units Superscript II Reverse Transcriptase (Invitrogen, Carlsbad, CA). Reactions were carried out in a single reaction for each experimental sample for 1 hr at 42°C followed by 15 min at 72°C. Briefly, PCR was carried out with 10 pMol/L each forward and reverse species specific and gene specific primers (TDI Integrated DNA Technologies, Coralville, IA). Also see Table S2), 1× SYBR Green (Bio-Rad, Hercules, CA) and 3.3 uL cDNA template in a total volume of 25 µl. The temperature parameters were as follows: annealing at 59°C for 40 cycles, followed by extension at 72°C for 30 sec. Each amplification was performed in triplicate. The thermal denaturation protocol at 95°C for 5 min was run at the end of the PCR to determine amplification of the specific products. The cycle number at which the reaction crossed an arbitrarily placed threshold (C_t_) was determined for each gene. Data were analyzed in an Excel spreadsheet using the 2^−ΔΔCt^ method to obtain the relative expression level and the HPRT as a normalization control in each sample. The relative amount of target = 2^−ΔΔCt^, where Ct is the threshold cycle for target amplification, ΔCt = Ct_ target gene_ – Ct_ internal reference_, and −ΔΔCt = ΔCt_ sample_ - ΔCt_ calibrator_. Statistical analysis was performed as previously published [23].

### Stimulation of CAMTA1 Expression in hMSCs Monocultures

Ionomycin was used to increase [Ca^2+^]_i_ and stimulate CAMTA1 expression in the stem cells. Stem cells were expanded in complete alpha MEM medium containing 10% fetal bovine serum and L-glutamine. Twenty four hrs prior to ionomycin exposure, all cells were changed to a medium containing 2% horse serum. Cells were stimulated for 6 hrs using a final concentration of 0.6 µMol/L ionomycin calcium salt (Sigma-Aldrich, St. Louis, MO). Control and ionomycin-treated cells were washed and harvested for RNA preparation. Remaining cells were washed extensively and allowed to recover in culture in fresh complete medium containing 2% horse serum. They were then harvested after 24 hrs of ‘recovery’. RNA was isolated using Qiagen’s RNEasy standard protocol for RNA isolation (Valencia, CA).

### Silencing of CAMTA1 in the Stem Cells

The effects of CAMTA1 on the expression of the cardiac transcription factors were investigated in loss-of-function-experiments:

#### A. Silencing CAMTA1 in hMSC monocultures

Human MSCs were transduced with GFP or CAMTA1 lentiviral particles (Santa Cruz, CA), used at vendor’s recommended concentrations for 6 hrs in 6 µg/ml Polybrene. All cells were then washed and maintained in culture for 24 hrs before Ca^2+^ stimulation with 0.6 µMol/L of ionomycin. All cells were then harvested for total RNA preparation

#### B. Silencing CAMTA1 in WB F344 stem cells co-cultured with cardiomyocytes

DsRed WB-F344 stem cells were maintained in culture as described previously [23]. They were transfected using Ambion’s siPORT Amine reagent (Austin, TX) with 30 nMol/L predesigned human CAMTA1 siRNAs pool or scrambled siRNA (Santa Cruz, CA) using the manufacturer’s recommended method. Transfected cells were washed 24 hrs later and cultured for an additional 48 hrs. The cells were then trypsinized, washed and seeded onto cardiomyocytes at a ratio of 1∶10 and allowed to continue in co-culture for an additional 48 hrs. The cells were then harvested by FACS for dsRed fluorescence and used for total RNA isolation (Qiagen RNEasy standard protocol). WB F344 cells used for control conditions were harvested for RNA directly from monocultures.

## Supporting Information

Table S1 **Change in gene expression in hMSCs co-cultured with cardiomyocytes (hMSCcc) for 4 days.**
(DOCX)

Table S2 **Primers used for qPCR.**
(DOCX)

Supporting Information S1 **Method sections describing Cardiac Microenvironment, Confocal Imaging of Calcium Signals, and Immunocytochemistry.**
(DOCX)

## References

[1] Wilmut I, Schnieke AE, McWhir J, Kind AJ, Campbell KHS (1997). Viable offspring derived from fetal and adult mammalian cells.. Nature.

[2] Gurdon JB, Melton DA (2008). Nuclear reprogramming in cells.. Science.

[3] Takahashi J (2007). Stem cell therapy for Parkinson’s disease.. Expert Rev Neurother.

[4] Takahashi K, Tanabe K, Ohnuki M, Narita M, Ichisaka T (2007). Induction of pluripotent stem cells from adult human fibroblasts by defined factors.. Cell.

[5] Takahashi K, Yamanaka S (2006). Induction of pluripotent stem cells from mouse embryonic and adult fibroblast cultures by defined factors.. Cell.

[6] Weintraub H, Tapscott SJ, Davis RL, Thayer MJ, Adam MA (1989). Activation of muscle-specific genes in pigment, nerve, fat, liver, and fibroblast cell lines by forced expression of MyoD.. Proc Natl Acad Sci USA.

[7] Choi J, Costa ML, Mermelstein CS, Chagas C, Holtzer S (1990). MyoD converts primary dermal fibroblasts, chondroblasts, smooth muscle, and retinal pigmented epithelial cells into striated mononucleated myoblasts and multinucleated myotubes.. Proc Natl Acad Sci USA.

[8] Davis RL, Weintraub H, Lassar AB (1987). Expression of a single transfected cDNA converts fibroblasts to myoblasts.. Cell.

[9] Hochedlinger K, Jaenisch R (2002). Monoclonal mice generated by nuclear transfer from mature B and T donor cells.. Nature.

[10] Yu J, Vodyanik MA, Smuga-Otto K, Antosiewicz-Bourget J, Frane JL (2007). Induced pluripotent stem cell lines derived from human somatic cells.. Science.

[11] Slack JM (2009). Metaplasia and somatic cell reprogramming.. J Pathol.

[12] Yamanaka S, Blau HM (2010). Nuclear reprogramming to a pluripotent state by three approaches.. Nature.

[13] Ieda M, Fu JD, Delgado-Olguin P, Vedantham V, Hayashi Y (2010). Direct reprogramming of fibroblasts into functional cardiomyocytes by defined factors.. Cell.

[14] Takeuchi JK, Bruneau BG (2009). Directed transdifferentiation of mouse mesoderm to heart tissue by defined factors.. Nature.

[15] Zhou Q, Brown J, Kanarek A, Rajagopal J, Melton DA (2008). In vivo reprogramming of adult pancreatic exocrine cells to beta-cells.. Nature.

[16] Vierbuchen T, Ostermeier A, Pang ZP, Kokubu Y, Sudhof TC (2010). Direct conversion of fibroblasts to functional neurons by defined factors.. Nature.

[17] Szabo E, Rampalli S, Risueno RM, Schnerch A, Mitchell R (2010). Direct conversion of human fibroblasts to multilineage blood progenitors.. Nature.

[18] Cesselli D, Beltrami AP, Rigo S, Bergamin N, D’Aurizio F (2009). Multipotent progenitor cells are present in human peripheral blood.. Circ Res.

[19] Dawn B, Bolli R (2005). Bone marrow cells for cardiac regeneration: the quest for the protagonist continues.. Cardiovasc Res.

[20] Kogler G, Sensken S, Wernet P (2006). Comparative generation and characterization of pluripotent unrestricted somatic stem cells with mesenchymal stem cells from human cord blood.. Exp Hematol.

[21] Pittenger MF, Martin BJ (2004). Mesenchymal stem cells and their potential as cardiac therapeutics.. Circ Res.

[22] Geraerts M, Verfaillie CM (2009). Adult stem and progenitor cells.. Adv Biochem Eng/Biotechnol.

[23] Muller-Borer BJ, Cascio WE, Esch GL, Kim HS, Coleman WB (2007). Mechanisms controlling the acquisition of a cardiac phenotype by liver stem cells.. Proc Natl Acad Sci USA.

[24] Malouf NN, Coleman WB, Grisham JW, Lininger RA, Madden VJ (2001). Adult-derived stem cells from the liver become myocytes in the heart in vivo.. Am J Path.

[25] Anderson PA, Muller-Borer BJ, Esch GL, Coleman WB, Grisham JW (2007). Calcium signals induce liver stem cells to acquire a cardiac phenotype.. Cell Cycle.

[26] De Koninck P, Schulman H (1998). Sensitivity of CaM kinase II to the frequency of Ca2+ oscillations.. Science.

[27] Bers DM (2008). Calcium cycling and signaling in cardiac myocytes.. Annu Rev Physiol.

[28] Grimm M, Brown JH (2010). Beta-adrenergic receptor signaling in the heart: role of CaMKII.. J Mol Cell Cardiol.

[29] Mani SK, Egan EA, Addy BK, Grimm M, Kasiganesan H (2010). beta-Adrenergic receptor stimulated Ncx1 upregulation is mediated via a CaMKII/AP-1 signaling pathway in adult cardiomyocytes.. J Mol Cell Cardiol.

[30] Mellstrom B, Naranjo JR (2001). Mechanisms of Ca(2+)-dependent transcription.. Curr Opin Neurobiol.

[31] Dolmetsch RE, Lewis RS, Goodnow CC, Healy JI (1997). Differential activation of transcription factors induced by Ca2+ response amplitude and duration.. Nature.

[32] Berridge MJ (2006). Calcium microdomains: organization and function.. Cell Calcium.

[33] Bading H, Ginty DD, Greenberg ME (1993). Regulation of gene expression in hippocampal neurons by distinct calcium signaling pathways.. Science.

[34] Ohba T, Watanabe H, Murakami M, Takahashi Y, Iino K (2007). Upregulation of TRPC1 in the development of cardiac hypertrophy.. J Mol Cell Cardiol.

[35] Frey N, McKinsey TA, Olson EN (2000). Decoding calcium signals involved in cardiac growth and function.. Nat Med.

[36] Wu H, Peisley A, Graef IA, Crabtree GR (2007). NFAT signaling and the invention of vertebrates.. Trends Cell Biol.

[37] Dolmetsch RE, Xu K, Lewis RS (1998). Calcium oscillations increase the efficiency and specificity of gene expression.. Nature.

[38] Doherty CJ, Van Buskirk HA, Myers SJ, Thomashow MF (2009). Roles for Arabidopsis CAMTA transcription factors in cold-regulated gene expression and freezing tolerance.. Plant Cell.

[39] Eckardt NA (2009). CAMTA proteins: a direct link between calcium signals and cold acclimation?. Plant Cell.

[40] Finkler A, Ashery-Padan R, Fromm H (2007). CAMTAs: calmodulin-binding transcription activators from plants to human.. FEBS Lett.

[41] Bouche N, Scharlat A, Snedden W, Bouchez D, Fromm H (2002). A novel family of calmodulin-binding transcription activators in multicellular organisms.. J Biol Chem.

[42] Song K, Backs J, McAnally J, Qi X, Gerard RD (2006). The transcriptional coactivator CAMTA2 stimulates cardiac growth by opposing class II histone deacetylases.. Cell.

[43] Crabtree GR, Schreiber SL (2009). SnapShot: Ca2+-calcineurin-NFAT signaling.. Cell 138(1): 210, 210 e211.

[44] Oh M, Dey A, Gerard RD, Hill JA, Rothermel BA (2010). The CCAAT/enhancer binding protein beta (C/EBPbeta) cooperates with NFAT to control expression of the calcineurin regulatory protein RCAN1–4.. J Biol Chem.

[45] Canellada A, Ramirez BG, Minami T, Redondo JM, Cano E (2008). Calcium/calcineurin signaling in primary cortical astrocyte cultures: Rcan1–4 and cyclooxygenase-2 as NFAT target genes.. Glia.

[46] Pandya K, Cowhig J, Brackhan J, Kim HS, Hagaman J (2008). Discordant on/off switching of gene expression in myocytes during cardiac hypertrophy in vivo.. Proc Natl Acad Sci USA.

[47] Pandya K, Kim HS, Smithies O (2006). Fibrosis, not cell size, delineates beta-myosin heavy chain reexpression during cardiac hypertrophy and normal aging in vivo.. Proc Natl Acad Sci USA.

[48] Nadri S, Soleimani M, Hosseni RH, Massumi M, Atashi A (2007). An efficient method for isolation of murine bone marrow mesenchymal stem cells.. Int J Dev Biol.

[49] Hatada S, Walton W, Hatada T, Wofford A, Fox R (2011). Therapeutic benefits in thalassemic mice transplanted with long-term cultured bone marrow cells.. Exp Hematol.

[50] Fukuhara S, Tomita S, Yamashiro S, Morisaki T, Yutani C (2003). Direct cell-cell interaction of cardiomyocytes is key for bone marrow stromal cells to go into cardiac lineage in vitro.. J Thorac Cardiovasc Surg.

[51] Kubo H, Berretta RM, Jaleel N, Angert D, Houser SR (2009). c-Kit+ bone marrow stem cells differentiate into functional cardiac myocytes.. Clin Transl Sci.

[52] Xu M, Wani M, Dai YS, Wang J, Yan M (2004). Differentiation of bone marrow stromal cells into the cardiac phenotype requires intercellular communication with myocytes.. Circ.

[53] Pijnappels DA, Schalij MJ, Ramkisoensing AA, van Tuyn J, de Vries AA (2008). Forced alignment of mesenchymal stem cells undergoing cardiomyogenic differentiation affects functional integration with cardiomyocyte cultures.. Circ Res.

[54] Kattman SJ, Huber TL, Keller GM (2006). Multipotent flk-1+ cardiovascular progenitor cells give rise to the cardiomyocyte, endothelial, and vascular smooth muscle lineages.. Dev Cell.

[55] Hutson MR, Zhang P, Stadt HA, Sato AK, Li YX (2006). Cardiac arterial pole alignment is sensitive to FGF8 signaling in the pharynx.. Dev Biol.

[56] Laflamme MA, Chen KY, Naumova AV, Muskheli V, Fugate JA (2007). Cardiomyocytes derived from human embryonic stem cells in pro-survival factors enhance function of infarcted rat hearts.. Nat Biotechnol.

[57] Kawano S, Shoji S, Ichinose S, Yamagata K, Tagami M (2002). Characterization of Ca(2+) signaling pathways in human mesenchymal stem cells.. Cell Calcium.

[58] Tomida T, Hirose K, Takizawa A, Shibasaki F, Iino M (2003). NFAT functions as a working memory of Ca2+ signals in decoding Ca2+ oscillation.. EMBO J.

[59] Colella M, Grisan F, Robert V, Turner JD, Thomas AP (2008). Ca2+ oscillation frequency decoding in cardiac cell hypertrophy: role of calcineurin/NFAT as Ca2+ signal integrators.. Proc Natl Acad Sci USA.

[60] Molkentin JD, Lu JR, Antos CL, Markham B, Richardson J (1998). A calcineurin-dependent transcriptional pathway for cardiac hypertrophy.. Cell.

[61] Williams RS, Rosenberg P (2002). Calcium-dependent gene regulation in myocyte hypertrophy and remodeling.. Cold Spring Harbor Symposia Quantitative Biology.

[62] Noseda M, Peterkin T, Simoes FC, Patient R, Schneider MD (2011). Cardiopoietic factors: extracellular signals for cardiac lineage commitment.. Circ Res.

[63] Ho L, Crabtree GR (2010). Chromatin remodelling during development.. Nature.

[64] Lessard JA, Crabtree GR (2010). Chromatin regulatory mechanisms in pluripotency.. Annu Rev Cell Dev Biol.

[65] Singhal N, Graumann J, Wu G, Arauzo-Bravo MJ, Han DW (2010). Chromatin-remodeling components of the BAF complex facilitate reprogramming.. Cell.

[66] Santourlidis S, Wernet P, Ghanjati F, Graffmann N, Springer J (2011). Unrestricted somatic stem cells (USSC) from human umbilical cord blood display uncommitted epigenetic signatures of the major stem cell pluripotency genes.. Stem Cell Res.

[67] Kidder BL, Palmer S, Knott JG (2009). SWI/SNF-Brg1 regulates self-renewal and occupies core pluripotency-related genes in embryonic stem cells.. Stem Cells.

[68] Koche RP, Smith ZD, Adli M, Gu H, Ku M (2011). Reprogramming factor expression initiates widespread targeted chromatin remodeling.. Cell Stem Cell.

[69] Crabtree GR, Olson EN (2002). NFAT signaling: choreographing the social lives of cells.. Cell.

[70] Henrich KO, Bauer T, Schulte J, Ehemann V, Deubzer H (2011). CAMTA1, a 1p36 tumor suppressor candidate, inhibits growth and activates differentiation programs in neuroblastoma cells.. Cancer Res.

[71] Battaglia A, Hoyme HE, Dallapiccola B, Zackai E, Hudgins L (2008). Further delineation of deletion 1p36 syndrome in 60 patients: a recognizable phenotype and common cause of developmental delay and mental retardation.. J Pediatr.

[72] Shapira SK, McCaskill C, Northrup H, Spikes AS, Elder FF (1997). Chromosome 1p36 deletions: the clinical phenotype and molecular characterization of a common newly delineated syndrome.. Am J Hum Genet.

[73] Vega RB, Rothermel BA, Weinheimer CJ, Kovacs A, Naseem RH, et al. Dual roles of modulatory calcineurin-interacting protein 1 in cardiac hypertrophy.. Proc Natl Acad of Sci USA.

[74] Alberts B, Bray D, Lewis J, Raff M, Roberts K (1989). Molecular Biology of the Cell; Alberts B, editor.. New York & London: Garland Publishing, Inc.

[75] Olson EN (2004). A decade of discoveries in cardiac biology.. Nat Med.

[76] Labovsky V, Hofer EL, Feldman L, Fernández Vallone V, García Rivello H (2010). Cardiomyogenic differentiation of human bone marrow mesenchymal cells: Role of cardiac extract from neonatal rat cardiomyocytes.. Differentiation.

[77] Li X, Zhu L, Yang A, Lin J, Tang F (2011). Calcineurin-NFAT signaling critically regulates early lineage specification in mouse embryonic stem cells and embryos.. Cell Stem Cell.

