# Supplementary material for: Calcium Dependent CAMTA1 in Adult Stem Cell Commitment to a Myocardial Lineage
Source: PLoS One. 2012 Jun 8;7(6):e38454. doi: 10.1371/journal.pone.0038454 (PMC3371086; doi:10.1371/journal.pone.0038454)
Supplement: Table S1 — Change in gene expression in hMSCs co-cultured with cardiomyocytes (hMSCcc) for 4 days. (DOCX) [file pone.0038454.s001.docx]

***Supporting Information Table S1***

Change in gene expression in hMSCs co-cultured with cardiomyocytes (hMSCcc) for 4 days

| **Gene symbol** | **Common Name** | **hMSC vs. hMSCcc**  **Fold-Change** | **Statistical Significance**  **p value** |
| --- | --- | --- | --- |
| SMARCA2 | BRM;SWI2;SNF2 | 0.635 | 0.0366 |
| SMARCB1 | SNF5;BAF47 | 0.659 | 0.0323 |
| SMARCE1  SMARCE1 | SMARCE1  BAF57 | 0.489  0.678 | 0.0313  0.0264 |
| SMARCA4 | SWI2;SNF2;BRG1 | 0.585 | 0.0117 |
|  |  |  |  |
|  |  |  |  |
|  |  |  |  |
|  |  |  |  |
|  |  |  |  |
|  |  |  |  |
|  |  |  |  |
