# Supplementary material for: Calcium Dependent CAMTA1 in Adult Stem Cell Commitment to a Myocardial Lineage
Source: PLoS One. 2012 Jun 8;7(6):e38454. doi: 10.1371/journal.pone.0038454 (PMC3371086; doi:10.1371/journal.pone.0038454)
Supplement: Table S2 — Primers used for qPCR. (DOCX) [file pone.0038454.s002.docx]

***Supporting Information Table S2:*** Primers used for qPCR

**Human mRNA**

| ***HPRT*** | Forward Primer: Reverse Primer: | 5'-ACCCTTTCCAAATCCTCAGC-3'  5’-TCCTCCTCCTGAGCAGTCA-3' |
| --- | --- | --- |
| ***CAMTA1*** | Forward Primer: Reverse Primer: | 5'-CAGCTCACTCTGTCTGATCA-3'  5'-ATAAGGATGGCAGCCTGTGT-3' |
| ***CAMTA2*** | Forward Primer: Reverse Primer: | 5'- CTCCTGCTCTTAGCACCATCAC-3'  5'-AGACACAGGAGTAATGCTCGGC-3' |
| ***RCAN1*** | Forward Primer: Reverse Primer: | 5'-GAGTCCCTCTTTAGGACGTATGACTGACTCGTGCTGCCCTCC ProbProbe:A-3'ods or _____________________________________________________________________________________-3'  5'-GCTTCCTATGTGTAAGGTCTGAGC-3' |
| ***RCAN3*** | Forward Primer: | 5’-CTGTTTCCAAATTGGGACCAG-3’ |
|  | Reverse Primer: | 5’-CAGACATGAACCACCACGCT-3’ |
| ***NFATc2*** | Forward Primer: | 5’-GAGCTTCATTGACTGCGAGATG-3’ |
|  | Reverse Primer: | 5’-GTCATACGTCCTAAAGAGGGACTC-3’ |
| ***Gata4*** | Forward Primer: Reverse Primer: | 5’-GTTCACCGTGTCACACATAGAG -3’  5’- GACTCTGTCCACTTACACTCTC-3’ |
| ***Mef2C*** | Forward Primer: Reverse Primer: | 5’-CTCAGAGTTCAAATCTCTCCCTGC-3’  5’-TACGGTCTCTAGGAGGAGAAACAG-3’ |
| ***Myocardin*** | Forward Primer: | 5’-CACAGCCTCCATCCTATGAA-3’ |
|  | Reverse Primer: | 5’-GGAGTTCATCCATCTGCTGA-3’ |
| ***Nkx2.5*** | Forward Primer: | 5’-GACCCTAGAGCCGAAAAGAA-3’ |
|  | Reverse Primer: | 5’- CTCTGTCTTCTCCAGCTCCA-3’ |
|  |  |  |
| ***Tbox5*** | Forward Primer: Reverse Primer: | 5'-CCTTCTCCAATATCACGTGCTCAG-3'  5'-GAATAAGATAGGATGCCAGGGAGC-3' |
| ***cTnT*** | Forward Primer: Reverse Primer: | 5'-TCACATAGAAGCCTAGCCCA-3'  5'-ATGTCAGACATGGTCTCTGC-3' |
| ***βMHC*** | Forward Primer: | 5’-TGTGTCACCGTCAACCCTTA-3’ |
|  | Reverse Primer: | 5’-TGGCTGCAATAACAGCAAAG-3’ |
| ***Cx43*** | Forward Primer: | 5’-ATGAGCAGTCTGCCTTTCGT-3’ |
|  | Reverse Primer: | 5’-TCTGCTTCAAGTGCATGTCC-3’ |

**Rat mRNA**

| ***HPRT*** | Forward Primer: Reverse Primer: | 5'-GCTGACCTGCTGGATTACATTA-3'  5’-CCACTTTCGCTGATGACACAA-3' |
| --- | --- | --- |
| ***CAMTA1*** | Forward Primer: Reverse Primer: | 5'-GGACCAGTGTAGTAAAGGAC-3'  5'-CATGTTCACCTGAATATCATCC-3' |
| ***CAMTA2*** | Forward Primer: Reverse Primer: | 5'- CTTTCCTAGTGAACTGCCCT-3'  5'-TCTGGATGACTCGTGCTG-3' |
| ***RCAN1*** | Forward Primer: Reverse Primer: | 5'- CAGGAGAGAAGTACGAGCTACA TGACTCGTGCTGCCCTCC ProbProbe:A-3'ods or _____________________________________________________________________________________-3'  5'- CTTCATTCTCTCCATCTCCTCC -3' |
| ***RCAN3*** | Forward Primer: | 5’-CCAGTGATCAACTATGACCTCCTC-3’ |
|  | Reverse Primer: | 5’-GTGTCTTCTTCCTCTTCGGTTTCG-3’ |
| ***NFATc2*** | Forward Primer: | 5’-GAGTGAAGTGAACAAGGCTGTC-3’ |
|  | Reverse Primer: | 5’-GAGAGTCCAAAGATGCTCCCTTAG-3’ |
| ***Gata4*** | Forward Primer: Reverse Primer: | 5’-CATGTGTCCCATACCTGATCTGAC -3’  5’- TACAGAGGGTAGGAGATGTTCAGG-3’ |
| ***Mef2C*** | Forward Primer: Reverse Primer: | 5’-CTCAGAGTTCAAATCTCTCCCTGC -3’  5’-TACGGTCTCTAGGAGGAGAAACAG -3’ |
| ***Myocardin*** | Forward Primer: | 5’-CCAGCCCCCATCCTATGAA -3’ |
|  | Reverse Primer: | 5’-GGAGTTCGTCGTCCATCTGCTGA-3’ |
| ***Nkx2.5*** | Forward Primer: | 5’-TGACCCTGACCCCGCCAA -3’ |
|  | Reverse Primer: | 5’- GTCTCGGCTTTGTCCAGCT-3’ |
|  |  |  |
| ***Tbox5*** | Forward Primer: Reverse Primer: | 5'-GAGTTACACAGGATGTCTCGGATG-3'  5'-GACACCATTCTCACACTGGTACTG-3' |
| ***cTnT*** | Forward Primer: Reverse Primer: | 5'-ATTCTGGCAGAGAGGAGGAA-3'  5'-GTGGATACTCTGCCATAGCT-3' |
| ***βMHC*** | Forward Primer: | 5’-TGGCACCGTGGACTACAATA-3’ |
|  | Reverse Primer: | 5’-TACAGGTGCATCAGCTCCAG-3’ |
| ***Cx43*** | Forward Primer: | 5’-TCCTTGGTGTCTCTCGCTTT-3’ |
|  | Reverse Primer: | 5’-GAGCAGCCATTGAAGTAGGC-3’ |
