# Supplementary material for: Calcium Dependent CAMTA1 in Adult Stem Cell Commitment to a Myocardial Lineage
Source: PLoS One. 2012 Jun 8;7(6):e38454. doi: 10.1371/journal.pone.0038454 (PMC3371086; doi:10.1371/journal.pone.0038454)
Supplement: Supporting Information S1 — Method sections describing Cardiac Microenvironment, Confocal Imaging of Calcium Signals, and Immunocytochemistry. (DOCX) [file pone.0038454.s003.docx]

***Supporting Information S1***

**Methods**

*Cardiac Microenvironment*

A cardiac microenvironment was generated using neonatal rat cardiomyocytes. These were isolated from the hearts of 1 day-old Sprague-Dawley rats in accordance with accepted guidelines for the care and treatment of experimental animals. Neonatal cardiomyocytes were isolated, plated on laminin-coated cover slides and grown in Richter’s medium (Irvine Scientific, Santa Ana, CA) supplemented with 10% fetal calf serum. Cardiomyocyte cultures were maintained for 48 hours before stem cells were added. Approval of the procedure to isolate neonatal rat cardiomyocytes was obtained from the Institutional Animal Care and Use Committee of Duke University, protocols A279-07-10 and A223-10-09.

*Confocal Imaging of Calcium Signals*

DsRed fluorescent hMSCs co-cultured with heart cells, were incubated in the calcium sensitive fluoroprobe, fluo-4 AM (5 µmol/liter; Molecular Probes, Eugene, OR) for 30 min at 20°C and subsequently washed several times with dye-free, phenol-red free Richter’s medium plus 2% horse serum before measurements were recorded. A three dimensional (3D) Z stack confocal fluorescence image was acquired to record cell position, alignment, and geometry with a LSM510 inverted laser-scanning confocal microscope with high-resolution optics (X63/1.4 N.A. oil immersion objective and environmental chamber; Carl Zeiss Microimaging, Thornwood, NY). The line scan mode with an excitation wavelength of 488 nm and an emission filter wavelength of 515 ± 15 nm were used for quantitative analysis of [Ca^2+^] signals. A line spanning a neonatal cardiomyocyte and an adjacent hMSC was defined, and fluorescence emission was collected at a rate of 1.92 ms per 1,024-pixel line. The location and origin of each Ca^2+^ recordings were verified with the confocal 3D Z stack image. Ca^2+^ recordings reported are from hMSCs and cardiomyocytes that were NOT overlapping or on top of other cardiomyocytes. The resulting time series images were analyzed with Image J software. Fluorescence signals were normalized by dividing the cell fluorescence (*F*) by cell fluorescence recorded before stimulation/contraction (*F*o).

*Immunocytochemistry*

Anti alpha-actinin (Sigma, (1:200); mouse monoclonal anti-cardiac troponin T antibody Mab13-11. [23]); anti CAMTA1 antibody (Santa Cruz Biochemicals, Santa Cruz, CA); anti NFAT2c antibody (Santa Cruz) were used. Cells were incubated with anti-alpha actinin (dilution 1:200; Sigma), mouse anti-cardiac troponin T (MAB13-11, 1:1000, Sigma), goat anti-CAMTA1 or NFATc2 (both at 1:50 dilution, Santa Cruz). antibodies used at dilutions proposed by the vendors (Invitrogen or Rockland Immunochemicals). Secondary antibodies were obtained from Invitrogen and/or Rockland Immunochemicals (Gilbertsville, PA) using dilutions proposed by the vendors. Nuclei were labeled with 4,6′-diamidino-2-phenylindole (DAPI; Invitrogen). Images were taken using laser confocal microscopy as noted in figure legends (Zeiss LSM 510 Axiovert). For all staining, samples were fixed with 2-4% paraformaldehyde prior to blocking in 10% Blocking Buffer (Pierce) and permeabilized using 0.2% Triton X-100 as previously described (24).
